# Factor structure and construct validity of the short form of managing the emotions of others (MEOS-SF) scale in the Chinese sample

**DOI:** 10.1371/journal.pone.0249774

**Published:** 2021-04-15

**Authors:** Qianglong Wang, Zhuo Zhang, Ping Song, Zhenbiao Liu, Qingyun Zhang, Anthony A. Vivino, Bo Yang, Ping Hu

**Affiliations:** 1 School of Foreign Languages, Taishan University, Tai’an, Shandong, China; 2 School of Criminal Justice, China University of Political Science and Law, Beijing, China; 3 School of Sociology, China University of Political Science and Law, Beijing, China; 4 Criminal Investigation and Counter-Terrorism College, Criminal Investigation Police University of China, Shenyang, Liaoning, China; 5 Department of Psychology, University of Maryland, College Park, MD, United States of America; 6 The Department of Psychology, Renmin University of China, Beijing, China; 7 The Laboratory of Department of Psychology, Renmin University of China, Beijing, China; Universitat de Valencia, SPAIN

## Abstract

**Objectives:**

Emotional manipulation is an important strategy in social interaction. The English version of MEOS-SF has been developed to make the measurement of such manipulation ability more efficient. The purpose of the current study was to assess the psychometric properties of the Chinese version of MEOS-SF.

**Methods:**

Explore factor analysis and Confirmatory factor analysis were adopted to examine the Chinese version of the MEOS-SF factor structure in 645 Chinese participants (mean age = 24.68 ± 6.01 years) recruited online.

**Results:**

Factor analysis supported a new three-factor model that included Conceal, Prosocial, and Non-prosocial, different from the original English MEOS-SF. Enhance and Divert merged to Prosocial factor while Worsen and Inauthentic merged to Non-prosocial factor because both prosocial and non-prosocial pairs had similar objectives, which would be perceived as the same thing by people in Eastern culture. As expected, MEOS-SF factors were found to be correlated with the Big Five, psychopathy, narcissism, Machiavellianism, and trait EI.

**Conclusions:**

Our results suggested that the Chinese version of MEOS-SF had acceptable psychometric properties and could be used to assess emotional manipulation.

## Introduction

Effective interpersonal manipulation needs individuals to own an ability to perceive the emotion expectation and expression of others accurately and predict their response to stimuli [1,2]. Interpersonal manipulation is closely associated with dark personalities such as psychopathy, Machiavellianism, and narcissism [3–7]. People who have these dark personalities may deploy different strategies for manipulating others’ emotions for self-serving intentions, such as to induce individuals to behave in a particular way or worsen someone’s feeling. Due to the lack of useful measurement of emotion manipulation, it is necessary to develop a useful tool to assess emotional manipulation’s underlying traits and its association with dark personalities. Therefore, the Managing the Emotions of Others Scale (MEOS) was developed as a multidimensional self-report scale to assess the individual difference in interpersonal emotion management [8].

The original MEOS version is in English, and six factors are extracted from 58 items. One prosocial pair factor (Enhance, Divert) and one non-prosocial pair factor (Worsen and Inauthentic) are core factors of MEOS used to capture the approaches of managing others’ emotions. The two prosocial factors represent mood-improvement, Enhance is described as offering to help or reassure and to express understanding of others, and Divert is described as changing another’s low or bad mood by using humor or arranging enjoyable activities. Two non-prosocial factors are related to mood-worsening. The items within Worsen contain the approach of using criticism to undermine others’ confidence. Inauthentic is related to emotional expression tactics such as flattery, sulking, or disingenuous niceness to influence other persons’ emotions. The other two factors are Conceal and Poor skills. The former represents the interpersonal emotion management ability to hide one’s emotion and not be detected by others. The latter represents the reduced ability to influence another’s emotion. However, because of unsatisfactory psychometric properties, the Poor Skills subscale items were removed in the Mandarin version of MEOS and the English version MEOS-SF [9,10]. Due to practical needs, a short form of MEOS has also been developed to meet the research design needs and keep participants’ positive interest when completing the scale [9]. However, the psychometric properties of MEOS-SF have not been examined in Chinese culture.

Associations between MEOS and personality have been found. Enhance and Divert had a positive, and Worsen and Inauthentic had a negative association with Agreeableness. Moreover, Worsen and Inauthentic were positively correlated with the Dark Triad, a group of personality traits including psychopathy, Machiavellianism, and narcissism [8,11]. Managing the emotions of others is also regarded as an essential part of emotional intelligence (EI) and has been broadly studied in the area of emotion regulation [12,13]. The association between MEOS and EI has also been examined that Enhance and Divert were positively correlated with trait EI while Worsen and Inauthentic had a negative correlation with trait EI [8]. In addition, trait EI has also been found to promote manipulative behaviors. High EI males may have fewer delinquent offenses and a high level of a prosocial (Enhance) approach to influence others, but women with high trait EI had manipulative relational behaviors [14].

This study aims to examine the translation and verification of the reliability and validity of MEOS-SF in a Chinese sample. This verification of a Chinese version of the MEOS-SF would increase the efficiency of research regarding emotional manipulation in Eastern culture, saving time and ensuring participants’ concentration. Moreover, the Chinese version of MEOS-SF will continue to expand the applicability of relevant research results in western populations.

We plan to test the MEOS-SF association with the Big Five personality scale, a trait EI scale, and three independent scales related to dark personalities (psychopathy, narcissism, Machiavellianism). According to previous researches in MEOS [8–11,15], dark personalities were expected to have positive relationships with non-prosocial factors and negative relationships with prosocial factors. EI and the Big Five personality were expected to have positive relationships with prosocial factors and negative relationships with non-prosocial factors. Due to cultural differences, a new factor structure was obtained in Chinses version of MEOS that Enhance and Divert were merged into a single factor [10]. Further exploration of this cultural difference in MEOS-SF among a broader Chinese range is also one of this study’s aims.

## Method

### Participants

The current data was collected in Beijing. Participants (N = 716) were adults recruited online by advertisement. Of the 716 participants, 71 were removed from the current study because of missing data. The final sample was 645 participants. The age of all participants ranged from 18 to 56 years (*M* = 24.68, *SD* = 6.01). Among these participants, 446 were men (69.19%), 199 were women (30.85%). Ninety-four participants had an education level of primary school (14.55%), 175 had an education level of junior school (27.08%), 111 had an education level of senior high school (17.18%), and 258 had an education level of university degree (39.93%), and 8 participants did not fill the education information. The major of participants were of Han ethnicity (95%), and the rest were ethnic minorities. We used Exploratory factor analysis (EFA) and Confirmatory factor analysis (CFA) to gain a MEOS-SF cross-validation model. In order to find a robust MEOS-SF factorial structure, EFA and CFA should be conducted in different samples [16]. So participants were randomly divided into Sample 1 (n = 322) and Sample 2 (n = 323).

### Procedure

Participants completed all the research scales on a website at times and locations of their choosing. Before filling in the scales, informed consent information appeared on the screen, and if the participants decided to agree to participate in our study, the task continued. It was also possible to opt-out voluntarily. The consent included information about researchers, research goals, research content (i.e., the items in scales were used to assess their personality, emotional intelligence, and the ability to manage others’ emotions), potential risks, reward, voluntary declaration, and Data Confidentiality Statement. After completing the task, 10 Yuan (about 1.3 USD) was given to them as a reward. This study was approved by the Institutional Research Ethics Committee of China University of Political Science and Law (2019072001).

### Measures

#### Managing the Emotions of Others Scale- Short Form (MEOS-SF)

The MEOS-SF consists of 30 items, each on an item ranging from 1 (strongly disagree) to 5 (strongly agree). The items of MEOS-SF in the present study were derived from the version of Austin’s publication in 2018 [9]. Five factors, mood-enhancing (Enhance), Enhance another’s mood by diversion (Divert), mood worsening (Worsen), use of inauthentic ways for self-serving intentions (Inauthentic), and concealing emotions from others (Conceal), are included in MEOS-SF. Each factor has six items. The Chinese version of MEOS-SF was translated into Chinese and back-translation to English by students major in English linguistics to ensure the consistency of Chinese and English meanings. All translation steps followed the standard procedure suggested by Brislin [17].

#### Trait Emotional Intelligence Questionnaire-Short Form (TEIQue-SF)

TEIQue-SF is designed to measure the global trait of emotional intelligence [18]. TEIQue-SF contains 30 items and uses a Likert-style response, ranging from 1 (completely disagree) to 7 (completely agree). The Chinese simplified version of TEIQue-SF was used in the current study (see http://psychometriclab.com/translations-of-teique/). In the current research, Cronbach’s alpha was.78.

#### Neuroticism Extraversion Openness Five-Factor Inventory (NEO-FFI)

NEO-FFI is developed by Costa and McCrae [19] to measure the fundamental five personality factors, Neuroticism (N), Agressable (A), Conscientiousness (C), Extraversion (E), and Openness (O). NEO-FFI consists of 60 items, ranging from 1 (completely disagree) to 5 (completely agree). The Chinese version of the NEO-FFI had excellent reliability and validity [20]. In the current research, Cronbach’s alpha was .66 (N), .60 (E), .62 (O),.63 (A) and .62 (C), respectively.

#### Levenson’s Self-report Psychopathy Scale (LSRP)

LSRP is a self-report scale widely used to measure psychopathic traits. LSRP consists of two dimensions, primary psychopathy (16 items) and secondary (10 items) psychopathy [21], and its response ranged from 1 (strongly disagree) to 4 (strongly agree). The Chinese version of LSRP was applied in the current studies [22]. The Cronbach’s alpha was .72 (primary) and .61 (secondary).

#### Machiavellianism (Mach IV)

Mach IV is a 20-item scale to assesses the personality of Machiavellianism [23]. It is a five-point Likert scale, ranging from 1 (strongly disagree) to 5 (strongly agree). The Chinese version was used in the current study [24]. The Cronbach’s alpha was .57.

#### Narcissistic Personality Inventory (NPI-40)

NPI-40 scale has 40 forced-choice items to assess grandiose narcissism [25]. Participants should choose between two statements that closest to their feelings. A Chinese version of NPI-40 was adopted in the current study [26]. Cronbach’s alpha was .82 in the current study.

### Statistical analysis

EFA was performed by SPSS23.0 to examine the dimensions underlying the Chinese MEOS-SF. Factors loadings≥0.4 were considered adequate. Alpha coefficients were analyzed to assess internal consistency for subscales scores in MEOS-SF. CFA was conducted in AMOS 24.0. Multiple fit indices were calculated: chi-square, the Tucker-Lewis index (TLI), the goodness-of-fit (GFI), the comparative fit index (CFI), and the root-mean-square error of approximation (RMSEA). According to general practice, each index’s acceptable standard is RMSEA≤0.1 [27], GFI and CFI, and TLI≥0.9 [28]. The zero-order correlations of the MEOS-SF with personality and trait EI were conducted to examine the validity coefficients.

## Results

### Descriptive statistics

Descriptive statistics for all measures are presented in Table 1. The Cronbach’s alpha of Conceal was lowest (.66), and the highest was Worsen (.81). The prosocial factors (Enhance and Divert) and non-prosocial factors (Worsen and Inauthentic) that both had the strongest positive correlations in the Chinese translation of MEOS-SF (see Table 2).

**Table 1:** Means (SD), Skewness, and Kurtosis for the MEOS-SF.

Scale	M (SD)	Skewness (*SE*)	Kurtosis (*SE*)	*N* of items
**MEOS-factors**				
MEOS-Conceal	20.35 (4.31)	-.53 (.09)	.58 (.19)	6
MEOS-Enhance	22.26 (4.03)	-.58 (.09)	.86 (.19)	6
MEOS-Divert	22.26 (4.42)	-.51 (.09)	.29 (.19)	6
MEOS-Worsen	16.67 (5.38)	-.10 (.09)	-.75 (.19)	6
MEOS-Inauthentic	16.59 (5.08)	-.18 (.09)	-.55 (.19)	6
MEOS-Total	98.13 (13.26)	-.44 (.09)	1.79 (.19)	30
**NEO-factors**				
NEO-Neuroticism	37.68 (9.86)	-.13 (.09)	-.70 (.19)	12
NEO-Extraversion	39.33 (6.72)	-.01 (.09)	.77 (.19)	12
NEO-Openness	38.92 (5.84)	-.01 (.09)	-.53 (.19)	12
NEO-Agreeableness	37.66 (6.98)	-.11 (.09)	-.11 (.19)	12
NEO-Conscientiousness	39.38 (7.46)	.11 (.09)	.26 (.19)	12
NEO-Total	192.97 (20.13)	-.08 (.09)	.63 (.19)	60
**LSRP-factors**				
LSRP-Primary	41.92 (5.76)	-.05 (.09)	-.90 (.19)	16
LSRP-Secondary	24.47 (3.57)	.39 (.09)	-.21 (.19)	10
LSRP-Total	66.49 (7.76)	-.04 (.09)	-.51 (.19)	26
**NPI-Total**	23.13 (8.33)	-.27 (.09)	-.03 (.19)	34
**TEIQue-SF-total**	131.40 (20.47)	.44 (.09)	-.32 (.19)	30
**Mach IV-Total**	54.47 (10.59)	-.13 (.09)	-.02 (.19)	20

*N = 645.

**Table 2:** Alpha reliabilities and intercorrelations for MEOS-SF factors.

	1	2	3	4	Cronbach’s α
Conceal			.		.66
Enhance	.27**				.68
Divert	.22**	.65**			.74
Worsen	.09*	-.11**	-.12**		.81
Inauthentic	.14**	-.12**	-.13**	.66**	.73

*p < .05

**p < .01.

### The factor structure of MEOS-SF

#### Exploratory factor analysis

An exploratory factor analysis (EFA) using principal axis factor with a Promax rotation was conducted to examine factors underlying the Chinese MEOS-SF in Sample 1 (n = 322). According to the scree plot, three factors were extracted from the MEOS-SF. The eigenvalues for the three factors were 4.81, 3.91, and 1.92. The KMO was .82. In the EFA matrix, Enhance and Divert rotated to factor1, Worsen and Inauthentic rotated to factor 2, and Conceal was factor 3. In the original version of MEOS [8], Enhance and Divert belong to prosocial factors while Worsen and Inauthentic belong to non-prosocial factors. Moreover, in Saklofske’s research with a Chinese sample, they also merged the Enhance and Divert into a single factor because of the high correlation of these two factors and their same objective in Chinese culture [10]. The non-prosocial factors also have the same target, although they were implemented differently: that people adopt emotional strategies to induce others’ negative moods to satisfy their purposes. These two pairs (Enhance and Divert; Worsen and Inauthentic) were also highly intercorrelated in the above results (r = .65, p<0.001; r = .66, p<0.001) as previous researches, extractions were considered reasonable.

Due to low (<0.40) and cross factor loading, seven items were removed from the MEOS-SF. Then the EFA was repeated on 23 items, and results showed that the KMO was .84, and three factors accounted for 42.09% of the variance. Pattern matrix elements are shown in Table 3, and item descriptions of MEOS-SF (23 items) are listed in Table 4.

**Table 3:** Standardized factor loadings of MEOS-SF (23 items).

	Factor 1 (prosocial)	Factor 2 (non-prosocial)	Factor 3
Enhance 1	.55		
Enhance 2	.45		
Enhance 3	.55		
Enhance 4	.54		
Divert 1	.57		
Divert 2	.57		
Divert 3	.55		
Divert 4	.54		
Divert 5	.57		
Divert 6	.57		
Worsen 1		.68	
Worsen 2		.66	
Worsen 3		.66	
Worsen 4		.63	
Worsen 5		.65	
Inauthentic 1		.48	
Inauthentic 2		.72	
Inauthentic 3		.50	
Inauthentic 4		.50	
Conceal 1			.62
Conceal 2			.52
Conceal 3			.47
Conceal 4			.58

**Table 4:** Item descriptions of MEOS-SF (23 items).

Items	Descriptions	Factors
**Conceal**		
Conceal 1	I often conceal feelings of anger and distress from others.	Conceal (4 items)
Conceal 2	When someone has made me upset or angry, I tend to downplay my feelings.	
Conceal 3	I don’t believe in telling others about my problems–I keep them to myself.	
Conceal 4	If someone tries to make me feel better when I am feeling low, I pretend to feel happier to please that person.	
**Enhance**		
Enhance 1	If someone is feeling anxious, I try to calm them down by talking with them.	Prosocial (10 items)
Enhance 2	When someone is anxious about a problem, I try to help them work out a solution.	
Enhance 3	If someone is anxious, I try to reassure them.	
Enhance 4	When someone is under stress I try to boost their confidence in their ability to cope.	
**Divert**		
Divert 1	If someone is angry, I try to divert their mood by being cheerful	
Divert 2	When someone is in a low mood I behave in a happy and cheerful way to make them feel better.	
Divert 3	When someone is in a bad mood I try to divert them by telling jokes or funny stories.	
Divert 4	When someone is unhappy I try to cheer them by talking about something positive.	
Divert 5	I sometimes use humor to try to lift another person’s mood.	
Divert 6	If someone is being awkward, I try to defuse the situation by being cheerful and pleasant.	
**Worsen**		
Worsen 1	I use anger to get others to do things that I want them to do.	Non-prosocial (9 items)
Worsen 2	I sometimes put someone down in public to make them feel bad.	
Worsen 3	I know how to make someone feel ashamed about something that they have done in order to stop them from doing it again.	
Worsen 4	I use criticism to make others feel that they should work harder.	
Worsen 5	If I don’t like someone’s behavior I make negative comments in order to make them feel bad.	
**Inauthentic**		
Inauthentic 1	I sometimes sulk to make someone feel guilty.	
Inauthentic 2	I sometimes sulk to get someone to change their behavior	
Inauthentic 3	If someone’s behavior has caused me distress, I try to make them feel guilty about it.	
Inauthentic 4	I sometimes use flattery to gain or keep someone’s good opinion.	

#### Confirmatory factor analysis

Maximum likelihood estimation was conducted in CFA to examine the factors structure of MEOS-SF in Sample 2 (n = 323). The standardized factor loadings all reached significance and ranged from 0.55 to 0.69. The mean loading was 0.60. Table 5 summarizes the fit indices of the Chinese version of MEOS-SF in the current sample. All fit indices other than TLI met the acceptable criteria.

**Table 5:** CFA model fit indices of MEOS-SF.

	χ^2^(df)	CFI	TLI	GFI	SRMR	RMSEA
MEOS-SF	445.63(249)	0.90	0.90	0.89	0.087	0.050

#### Reliability and validity

Internal reliability (Cronbach’s alpha) of the new three factors structure of MEOS-SF are shown in Table 6. Twenty-nine participants completed a retest of the MEOS-SF approximately two months later. The zero-order correlations were used to calculate the associations between Chinese MEOS-SF and external criteria scales. All the correlations were as expected. The internal reliability of Conceal had improved after removing two items. The other two factors were acceptable to good (.82 and .85). Besides, the correlations among the MEOS factors found that MEOS-Prosocial and MEOS-Non-prosocial were weak but significantly negative correlated (r = -.16, p<0.01), and Conceal was also correlated with Non-prosocial and Prosocial (r = .15, p<0.01; r = .20, p<0.01).

**Table 6:** Internal reliability and the bivariate correlations between MEOS-SF and other scales.

	MEOS-Conceal	MEOS-Prosocial	MEOS-Non-prosocial
NEO-Neuroticism	.15**	-0.04	.05
NEO-Extraversion	-.05	.34**	-.12**
NEO-Openness	.12**	.45**	-.14**
NEO-Agreeableness	.09*	.51**	-.44**
NEO-Conscientiousness	.05	.54**	-.25**
LSRP-Primary	-.01	-.30**	.49**
LSRP-Secondary	-.01	-.25**	.43**
LSRP-Total	-.01	-.33**	.56**
NPI-Total	.02	.15**	.28**
TEIQue-SF-total	.03	.49**	-.26**
Mach IV-Total	.06	-.39**	.52**
Cronbach’s alpha	.65	.82	.85
Test-Retest	.96**	.98**	.95**

*p < .05

**p < .01.

## Discussion

The purpose of this research was to examine the psychometric properties of the Chinese version of MEOS-SF, a self-report that measures respondents’ interpersonal emotion management ability. Results supported a three-factor model consisting of Conceal, Prosocial, and Non-prosocial that produced acceptable model fits. Reliability, including internal reliabilities and re-test reliabilities, were calculated and acceptable. Construct validity was supported by correlation results that were similar to previous studies [8–11,15]. Our findings revealed that the Chinese version of MEOS-SF could be regarded as a reliable and valid measure of interpersonal emotional manipulation in the Chinese population.

The EFA and CFA results revealed that the Chinese MEOS-SF possessed a stable three-factor structure with acceptable loading on each factor, which is inconsistent with the original five-factor English MEOS-SF [8]. In the current study, the Enhance/Divert pair rotated to the Prosocial factor, and Worsen/Inauthentic rotated to the Non-prosocial factor. The Enhance/Divert pair had been merged in a previous study [10] due to their shared objective to improve people’s moods. Because the goals of these two different interpersonal methods were the same, Eastern culture perceived them as the same strategy regardless of their differences [10]. Similarly, Worsen and Inauthentic were merged into the Non-prosocial because in Eastern culture’s perception, the strategies in these factors worsen others’ moods to serve personal goals and are not differentiated. Cultural differences were also found in social support: when others need help, Westerners will offer emotion-focused strategies while Easterners would offer both emotion-focused and problem-focused strategies [29]. In the MEOS, Enhance and Inauthentic are emotion-focused strategies, and Divert and Worsen are problem-focused, which indicates that the factors in the Enhance/Divert and Inauthentic/Worsen pairs may be less differentiated in Eastern culture. These results remind us that the differences between Eastern and Western cultures should be taken into account in future studies regarding emotional expression and regulation.

The correlations among the MEOS-SF factors correspond to the previous finding that Prosocial (Enhance/Divert) was significantly negatively correlated with Non-prosocial (Worsen/Inauthentic) [8,10,11]. However, the correlation size (r = -.16, p<0.001) was not large, meaning that these two factors are different in the Chinese sample and that people may hold two strategies at the same time to guide their behaviors. Nevertheless, this correlation was not in accordance with previous results in a Chinese sample [10], in which Enhance/Divert was negatively correlated with Worsen (-.57) while positively correlated with Inauthentic (.22). The contradiction between the two studies needs further exploration.

To provide initial evidence for its validity, we examined the correlations between the Chinese MEOS subscales and Big Five personality traits, psychopathy, narcissism, Machiavellianism, and trait EI. The Non-prosocial factor was positively correlated with narcissism, Machiavellianism, and psychopathy (primary and secondary), while the Prosocial factor was negatively correlated with these personalities. The Prosocial factor was found to have positive correlations with Openness, Agreeableness, and Conscientiousness, whereas the Non-prosocial factor was negatively correlated with these. The above associations between NEO dimensions and MEOS were also in line with previous results [8,10,11,15]. The associations of trait EI and MEOS-SF factors were positive with Prosocial and negative with Non-prosocial. That these two correlations (r = .49, p < 0.001; r = -.26, p<0.001) have medium effect sizes means that people are less likely to use both Prosocial and Non-prosocial methods to manipulate interpersonal emotion at the same time. Individuals with high EI tend to employ manipulation strategies that improve others’ moods, whereas those with low EI would do the opposite.

Overall, the general pattern of correlations provides evidence for the validity of the Chinese version of the MEOS-SF. Our translation of the MEOS-SF will help in-depth research regarding psychopathy and Machiavellianism in Eastern cultures because interpersonal manipulation is a core trait in these personality types [5,30,31] (Austin, Farrelly, Black, & Moore, 2007; Hare et al., 1989; Kraut & Price, 1976). Future work is needed to expand the sample size further to examine the factor structure and explore the coincidence between Chinese MEOS-SF results and relevant behavioral tasks assessing effect. For example, there is a close relationship between criminal groups and dark personalities, which may be potentially related to interpersonal manipulation. The role of Agreeableness and other NEO traits at play in EI and emotional manipulation remain to be examined in Chinese culture.

## Conclusions

This study is the first to analyze the factor structure and construct validity of MEOS-SF in the Chinese sample. Results of EFA and CFA suggest a successful translation from the English version into Chinese. After removing eight items because of their low factor loading, 23 items remain. Three factors are extracted, including Conceal, Prosocial (Enhance/Divert), and Non-prosocial (Inauthentic/Worsen). This new factor structure is different from the previous, but all acceptable psychometrics indexes suggest its fitness in Chinese culture. The Chinese version of MEOS-SF will contribute to future exploration of interpersonal emotional manipulation in the non-Western population.

## Supporting information

S1 File Chinese version of MOES-SF. (DOCX)

S2 File DOI of original scale. (DOCX)
