# Supplementary material for: Factor structure and construct validity of the short form of managing the emotions of others (MEOS-SF) scale in the Chinese sample
Source: PLoS One. 2021 Apr 15;16(4):e0249774. doi: 10.1371/journal.pone.0249774 (PMC8049282; doi:10.1371/journal.pone.0249774)
Supplement: S1 File — (DOCX) [file pone.0249774.s001.docx]

**人际情绪管理问卷**

|  | **非常**  **不赞同** | **有点**  **不赞同** | **不确定** | **有点赞同** | **非常赞同** |
| --- | --- | --- | --- | --- | --- |
| 1. 当我情绪低落的时候，如果有人对我好，我就假装变快乐以回应 | 1 | 2 | 3 | 4 | 5 |
| 2. 当他人情绪低落的时候，我会以快乐和愉悦的方式来使他们感觉变好 | 1 | 2 | 3 | 4 | 5 |
| 3. 我经常隐藏对别人的愤怒和失落 | 1 | 2 | 3 | 4 | 5 |
| 4. 当有人让我心烦或生气时，我倾向于冷处理 | 1 | 2 | 3 | 4 | 5 |
| 5. 如果有人表现得很尴尬，我会尝试通过欢快和愉悦来缓和这种情况 | 1 | 2 | 3 | 4 | 5 |
| 6. 当他人在处理棘手的事情时，我会鼓励他们，让他们确信他们应付得很好 | 1 | 2 | 3 | 4 | 5 |
| 7. 当他人感到愤怒时，我会尝试帮助他们洞察自身情绪 | 1 | 2 | 3 | 4 | 5 |
| 8. 我发怒使别人按我的想法做事 | 1 | 2 | 3 | 4 | 5 |
| 9. 我有时用幽默来提高别人的心情 | 1 | 2 | 3 | 4 | 5 |
| 10. 当他人不开心的时候，我试着通过谈论一些积极的事情来鼓励他们 | 1 | 2 | 3 | 4 | 5 |
| 11. 如果我想让别人为我做点什么，我先会尝试引起他们的同情 | 1 | 2 | 3 | 4 | 5 |
| 12. 当他人不开心时，我会尝试帮助他们用更积极的眼光来看待他们的处境 | 1 | 2 | 3 | 4 | 5 |
| 13. 我有时利用那些会引起对他人情绪反应的因素，来激怒他们 | 1 | 2 | 3 | 4 | 5 |
| 14. 我不会因信任而告诉别人我的问题——我会把它们藏在心里 | 1 | 2 | 3 | 4 | 5 |
| 15. 如果我不喜欢他人的行为，我会对他们做出负性的评价，使他们感觉糟糕 | 1 | 2 | 3 | 4 | 5 |
| 16. 我有时通过生气使别人改变他们的行为 | 1 | 2 | 3 | 4 | 5 |
| 17. 当他人不开心时，我会安抚他们，告诉他们事情会越来越好的 | 1 | 2 | 3 | 4 | 5 |
| 18. 我可以让他人感到焦虑，这样他们就会以特定的方式行动 | 1 | 2 | 3 | 4 | 5 |
| 19. 当他人心情不好时，我会讲笑话或有趣的故事来转移他们的注意力 | 1 | 2 | 3 | 4 | 5 |
| 20. 如果有人说或做了我不喜欢的事，我有时会生气 | 1 | 2 | 3 | 4 | 5 |
| 21. 我用愤怒的表现来激励别人 | 1 | 2 | 3 | 4 | 5 |
| 22. 如果有人生气了，我就试着用快乐的情绪来转移他们的情绪 | 1 | 2 | 3 | 4 | 5 |
| 23. 我有时夸大个人或健康问题，这是为了获得同情和避免做某件事 | 1 | 2 | 3 | 4 | 5 |
