# Supplementary material for: Factor structure and construct validity of the short form of managing the emotions of others (MEOS-SF) scale in the Chinese sample
Source: PLoS One. 2021 Apr 15;16(4):e0249774. doi: 10.1371/journal.pone.0249774 (PMC8049282; doi:10.1371/journal.pone.0249774)
Supplement: S2 File — (DOCX) [file pone.0249774.s002.docx]

Original scale: Development and Validation of Two Short Forms of the Managing the Emotions of Others (MEOS) Scale DOI: 10.3389/fpsyg.2018.00974

The MEOS-SF scale was published by Elizabeth J. Austin in an open-access article (Fronters in psychology) under a CC-BY license.
